# Longitudinal analysis of health status the first year after trauma in severely injured patients

**DOI:** 10.1186/s13049-020-00719-8

**Published:** 2020-04-20

**Authors:** Roos Johanna Maria Havermans, Mariska Adriana Cornelia de Jongh, Leonie de Munter, Koen Willem Wouter Lansink

**Affiliations:** 1 Brabant Trauma Registry, Network Emergency Care Brabant, Hilvarenbeekseweg 60, 5022 GC Tilburg, The Netherlands; 2 Department Trauma TopCare, ETZ hospital, Tilburg, The Netherlands; 3 Department of Surgery, ETZ hospital, Tilburg, The Netherlands

**Keywords:** Trauma, Severely injured, Health status, Prognostic factors over time

## Abstract

**Purpose:**

While survival rates after a trauma are increasing a considerable part of the trauma population are still at risk for both short and long term disabilities. Little is known about prognostic factors over time after a severe trauma. The aim of the present prospective cohort study was to examine trauma and patient related prognostic factors for a lower health status over time after a severe trauma.

**Methods:**

A multicentre prospective observational cohort study was conducted. Adult trauma patients with severe injuries (ISS ≥ 16) were included from August 2015 until November 2016 if admitted to one of the hospitals in Noord-Brabant (the Netherlands). Outcome measure was health status, measured by the EuroQol-5D (EQ-5D utility and EQ-Visual analogue scale) and the Health Utilities Index (HUI2 and HUI3) one week and one, three, six, and twelve months after injury. Patient and trauma characteristics were analysed as prognostic factors with linear mixed models. The effect of each prognostic factor over time was analysed by adding the interaction term between the prognostic factor and time point in a multivariable linear mixed model, adjusted for confounders. Additionally, the risk factors for problems in the EQ-5 dimensions of HS and cognition were analysed.

**Results:**

In total 239 severely injured patients participated. Pre-injury health status, hospital length of stay, ISS and comorbidities were significant prognostic factors for a lower health status. A younger age and extremity injury were prognostic factors for a lower health status until one month after trauma and unemployment before trauma and comorbidities six until twelve months after trauma. In the EQ-5 dimensions 44.1% remained problems in mobility, 15.3% in self-care, 46.4% in activity, 53.3% in pain, 32.5% in anxiety and 35.7% in cognition.

**Conclusions:**

Lower pre-injury health status, longer hospital length of stay, higher ISS, and comorbidities were significant prognostic factors for a lower health status during one year after a severe injury. A younger age and an extremity injury were short-term prognostic factors and unemployment before trauma and comorbidities were long-term prognostic factors. Even after twelve months patients in our population reported more problems in all EQ-5D dimensions when compared to the Dutch reference population.

## Background

Over the last several decades many studies have shown improved mortality rates in trauma centres [1–9]. These patients might be at risk of short and long-term disabilities [10–15]. Two years after injury only 23% of the severely injured patients returned to their pre-injury level of function and 70% resumed prior employment status [16, 17]. Several patient and trauma characteristics are associated with health status, a self-reported assessment for patients about their ability to function [18, 19], of trauma patients [20]. Significant differences are shown between Intensive Care Unit (ICU) and non-ICU patients, patients admitted to the ICU reported a significant lower physical function [21]. Besides, living alone, inability to return to work, comorbidities, low educational level, brain injury, spinal cord injury, lower extremity injury, and a higher Injury Severity Score (ISS) are shown to be associated with a lower post-injury health status [22–24].

In 2016 almost 4500 severely injured patients (Injury Severity Score (ISS) ≥ 16) were hospitalised in the Netherlands [25]. The group of severely injured patients is a heterogeneous group of patients. Both patient and trauma characteristics vary within various categories of injuries. The mortality of the severely injured patients was 16%, so 84% of the patients will survive and need to rehabilitate after a life-changing event [25]. Most studies about post-injury health status are retrospective in design. Although some studies examined the prognostic factors for a lower health status in severely injured patients little is known about the differences between short- and long-term prognostic factors. De Munter et al. (2019) [26] showed that pre-injury status is an important predictor of health status in the whole trauma population. However, it is unknown if these predictors are equal in the group of severely injured patients. The prognostic factors over time after a severe trauma should be investigated to improve understanding of the consequences of a severe injury.

The aim of the present prospective cohort study was to examine which trauma (e.g. ISS, injured body region) and patient related factors (e.g. ASA classification, age) are prognostic factors for a lower health status (EQ-5D utility, EQ-VAS, HUI2 and HUI3) in severely injured patients. Another aim is to gain insight into the development of the prognostic factors over time and the percentage of patients reporting disabilities in the EuroQol-5 dimensions including cognition over time after a severe trauma.

## Methods

### Study setting

**Fig. 1:**
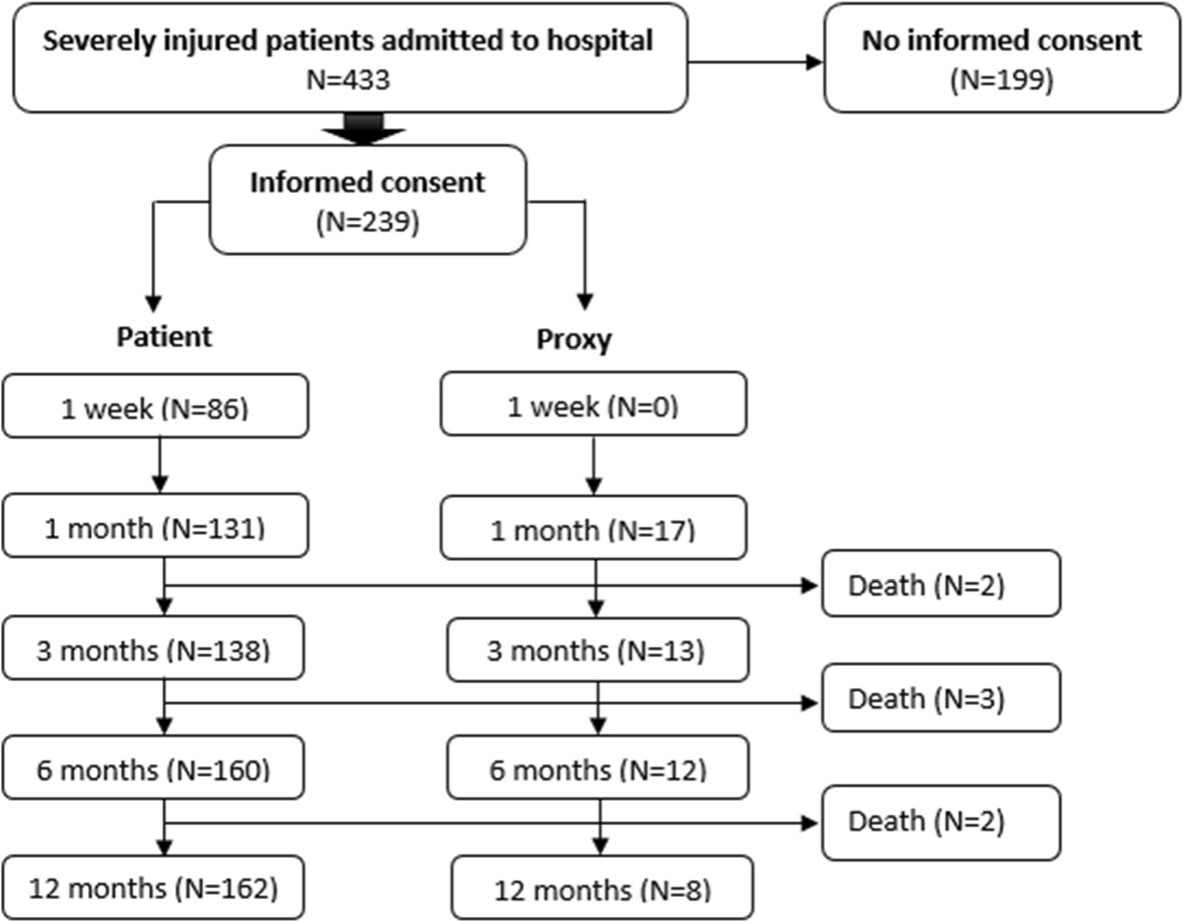
Flowchart of the number of included and excluded patients

The data of a cohort of severely injured patients were derived from the Brabant Injury Outcome Surveillance (BIOS) [27] study. Severely injured patients are defined as patients with an ISS ≥ 16 [25]. Adult patients (≥18 years) were included if they were admitted to an ICU or a ward in Noord-Brabant (the Netherlands) within 48 h after injury and who survived to hospital discharge between August 2015 and November 2016. Patients with pathological fractures or insufficient knowledge of the Dutch language were excluded. A total of 433 severely injured patients were asked to complete the questionnaires, 55.2% (*N* = 239) of them agreed to participate, see Fig. 1.

The study was approved by the Medical Ethics Committee (NL50258.028.14) and all participating patients or the proxy informants signed informed consent.

### Data collection - follow-up questionnaires

Data were collected by self-reported questionnaires by paper or electronic at one week, and one, three, six and twelve months after injury. If patients were discharged within one week after injury, questionnaires were sent by post. If the patient was still hospitalised after one week, questionnaires were distributed by a nurse or medical doctor.

- EuroQol-5D (EQ-5D), 3-level version [28, 29] - Measurement of health status in five dimensions: mobility, self-care, usual activities, pain/discomfort and anxiety/depression. A scoring algorithm is available for the EQ-5D by which each health status description can be expressed into a summary score [28]. This summary score ranges from 0 for death and 1 for full health. The validated EQ-5D questionnaire does not include cognitive disability. One additional question was added to cognition (“I have no/some/extreme problems with cognitive functioning, eg, memory, concentration, coherence, IQ”) [28] and will be used to describe the percentage of patients with cognitive limitations. The percentage of patients reporting problems in the EQ-5 dimensions in our population were compared with the Dutch reference population as described by Hoeymans et al.(2005) [30]. The EQ-5D has been used in various studies measuring health status [12, 23] and health related quality of life (HRQoL) in trauma patients [31, 32]. Many studies used the term HRQoL however measuring self-perceived health status [18].
- EuroQol-Visual analogue scale (EQ-VAS) - Measurement of health status, ranged from 0 as worst health state to 100 as best health state.
- Health Utilities Index (HUI) [33–35] - The results of the HUI questionnaires were converted by an algorithm into the levels of the complementary HUI2 and HUI3 classification system. HUI2 consists of seven dimensions with three to five levels: sensation, mobility, emotion, cognition, self-care, pain and fertility. HUI3 is mainly focusing on the primary functions (vision, hearing, speech, ambulation, dexterity, emotion, cognition and pain) with five to six levels. The HUI2 and HUI3 have been used in a large variety of clinical studies to measure health status [33–36].

All patients that responded to a questionnaire after one week or one month received a questionnaire about their pre-injury health status (EQ-5D pre-injury and EQ-VAS pre-injury). A combination of the HUI and EQ-5D has been found to be acceptable and valid in the trauma population [34, 37], therefore a combination should be used to define injury-related disability and health status [34, 38, 39]. Health status was defined as an individual’s level of function (physical, mental and social).

### Data collection - registry data

Prehospital data, trauma mechanism, injured body region,, diagnosis by AIS codes, injury severity and in-hospital medical procedures were obtained directly from the National Trauma RegistryInclusion criteria for the National Trauma Registry are hospitalisation within 48 h after a trauma, independent of their type or severity of injuries. Patient characteristics were extracted from the socio-demographic questions in the questionnaire. The Abbreviated Injury Scale (AIS-90, update 2008) [40, 41] was used to define the anatomical region and severity of separate injuries in detail and can be used to determine multiple injury. An AIS score of ≥3 was seen as a severe injured body region. The subgroups of head and face injuries and upper and lower extremity injuries were merged because of the small number of patients with a face and upper extremity injury (both subgroups consist of three patients). The ISS [42, 43] was used to assess overall trauma severity. To measure comorbidities, the American Society of Anaesthesiologists (ASA) classification system was used [44]. Patients with an ASA III and ASA IV classification were combined due to low prevalence.

Educational level was measured as the highest completed degree, certificate or diploma of education and was restructured in three categories: low educational level (primary education or preparatory secondary vocational education), middle educational level (university preparatory education, senior general secondary education or senior secondary vocational education) and high educational level (university of applied science or an academic degree).

### Statistical analysis

All analyses were conducted using IBM SPSS version 24 (Armonk, NY: IBM Corp, USA) and R version 3.4.0 (R Foundation for Statistical Computing, Vienna, Austria). Frequencies and descriptive statistics were calculated to provide an overview of the characteristics of the study population. Statistical test results were considered significant at a univariate level of *p* < 0.05. Patient characteristics were compared between responders and non-responders, with Mann-Whitney U tests (non-normal distributed continuous variables), independent t-test (normal distributed continuous variables) and Chi-square tests (categorical variables). Mean values with standard deviation will be presented for normally distributed data and median with interquartile range for not normally distributed data. Linear mixed models with random intercepts were used to examine health status over time with EQ-5D utility, EQ-VAS, HUI2 and HUI3 as outcome measurements. All completed measurements are included in the linear mixed models. All prognostic factors which met the Akaike Information Criterion (AIC) [45] in the univariate model for at least one of the four health status endpoints were included in the mixed models, both socio-demographic and injury-related characteristics.

To gain insight into the effect of prognostic factors over time during twelve months after trauma the interaction between the prognostic factors and time were examined. By changing the reference category of the time variable in the multivariable linear mixed model the main effect of the prognostic factor was calculated at each time point, adjusted for all other prognostic factors. The effect of the prognostic factor and its 95% confidence interval was visualised in Additional file 1. Variables resulting in a lower health status during the first three months after trauma were seen as short-term prognostic factors and variables resulting in a lower health status six until twelve months after trauma were seen as long-term prognostic factors.

The outcomes per EQ-5 dimension were dichotomised into problems (e.g. ‘I am confined to bed’ and ‘I have some problems in walking about’) versus no problems (e.g. ‘I have no problems in walking about’) [30]. Firstly, the odds ratio for problems in each dimension individually was analysed in a univariate longitudinal model. Secondly, the AIC was used to add variables in the generalized multiple linear mixed models.

Missing values for participants that completed the pre-injury assessment were imputed according to multiple imputation with 15 imputations and 5 iterations using the multivariate imputation by chained equations (MICE) procedure [46]. The missing follow-up EQ-5D utility scores, EQ-VAS scores, HUI2 and HUI3 for patients who died during the 12 months follow-up period were set to 0.

## Results

### Baseline characteristics

Responders were significant more often healthy (with an ASA I classification for 46.4% versus 32.5% and an ASA III/IV classification for 10.4% versus 20.6%) and a higher proportion of responders had an extremity injury compared to non-responders. The other variables did not differ between responders and non-responders. Within the patients with an extremity injury there were only three patients with an upper extremity injury.

Most patients were male 61.5% (*N* = 147) and the mean age was 56.3 years (SD 19.3), varying from 18 till 92 years (range 74). The median of ISS was 20 (Inter Quartile Range (IQR): 17–24) and the median of GCS was 15 (IQR: 13.5–15.0). The median of hospital length of stay (H-LOS) was 10 days (IQR: 5.0–17.8). From all patients, 43.5% (*N* = 104) of the patients were employed before trauma. These results are summarized in Table 1.

**Table 1:** Patient characteristics in the total cohort, the responders and the non-responders

	Total cohort	Responders	Non-responders	*p*-value
N (%)	433	239	194	
Age, mean (SD)	56.2 (19.9)	56.31 (19.3)	56.13 (20.7)	0.949
Male, n (%)	277 (64.0)	147 (61.5)	130 (67.0)	0.268
ISS, median [IQR]	19 [17–24]	20 [17–24]	19 [17–22.5]	0.321
GCS, median [IQR]^a^	–	15 [13.5–15.0]	–	–
H-LOS, median [IQR]	10 [5–17]	10 [5–17.75]	9 [4.75–17]	0.153
ICU admission, n (%)	276 (63.7)	159 (66.5)	117 (60.3)	0.406
ASA classification, n (%)				0.010
*ASA I*	174 (40.2)	111 (46.4)	63 (32.5)	
*ASA II*	155 (35.8)	83 (34.7)	72 (37.1)	
*ASA III/IV*	65 (15.0)	25 (10.4)	40 (20.6)	
*Missing*	39 (9.0)	20 (8.4)	19 (9.8)	
Region of injury with AIS ≥ 3, n (%)				
*Head or face*	219 (50.6)	111 (46.4)	108 (55.7)	0.056
*Thorax*	173 (40.0)	97 (40.6)	76 (39.2)	0.766
*Abdomen*	41 (9.5)	23 (9.6)	18 (9.3)	0.903
*Spine*	49 (11.3)	31 (13.0)	18 (9.3)	0.228
*Extremity*	76 (17.6)	50 (20.9)	26 (13.4)	0.041
Ventilation, n (%)	86 (19.9)	46 (19.2)	40 (20.6)	0.722
Multi person household, n (%)^a^	–	140 (58.6)	–	–
Employed, n (%)^a^	–	104 (43.5)	–	–

Legend: *N* Number, *SD* Standard deviation, *ISS* Injury Severity score, *GCS* Glasgow Coma Scale, *H-LOS* Hospital length of stay, *ICU* Intensive Care Unit, *ASA* American Society of Anaesthesiologists classification, *AIS* Abbreviated Injury Scale, *IQR* Interquartile range. ^a^Unknown in the non-responders

**Fig. 2:**
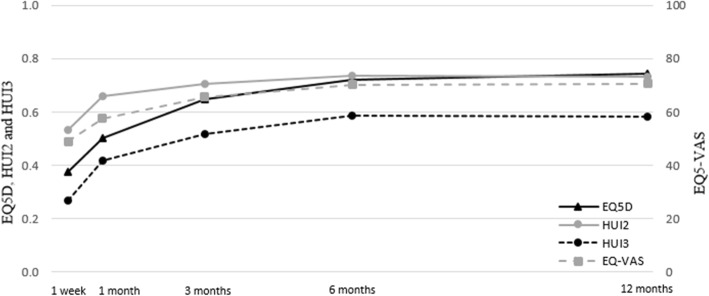
Mean utility scores of Health Status over time

Overall, the crude mean EQ-5D utility, EQ-VAS score and HUI scores increased over time, see Fig. 2. The largest increase of health status was found in the first month after trauma and the decline in percentage of patients with problems in the EQ-5 dimensions was largest between one month and three months.

### Health status: EQ-5D

In the adjusted model patient related prognostic factors for a significant lower EQ-5D utility score over time were a lower pre-injury EQ-5D score (β = 0.487; 95% CI: 0.289–0.686) and ASA III or ASA IV classification (β = − 0.137; 95% CI: − 0.257– − 0.016). Trauma related prognostic factors over time were longer H-LOS (β = − 0.005; 95% CI: − 0.008– − 0.002) and a higher ISS score (β = − 0.010; 95% CI: − 0.017– − 0.004). Patients with an abdomen injury scored significant higher compared to patients without an abdomen injury (β = 0.138; 95% CI: 0.012–0.264). See Table 2 for an overview of the prognostic factors.

**Table 2:** Longitudinal analysis of health status during one year after a severe injury

	EQ-5D beta (95% CI)	EQ-VAS beta (95% CI)	HUI2 beta (95% CI)	HUI3 beta (95% CI)
Age	0.001 (− 0.001–0.004)	−0.008 (− 0.181–0.166)	0.001 (− 0.001–0.003)	0.001 (− 0.002–0.004)
ASA II^1^	− 0.004 (− 0.076–0.068)	1.559 (− 3.936–7.054)	− 0.022 (− 0.086–0.042)	−0.029 (− 0.122–0.064)
ASA III/IV^1^	− 0.137 (− 0.257– − 0.016)*	−6.913 (− 16.540–2.715)	−0.176 (− 0.289– − 0.063)*	−0.213 (− 0.376– − 0.049)*
Female gender	0.004 (− 0.063–0.070)	− 2.481 (− 7.563–2.600)	−0.031 (− 0.089–0.027)	−0.015 (− 0.101–0.070)
Employed	0.003 (− 0.079–0.084)	−3.184 (− 9.601–3.234)	0.041 (− 0.034–0.116)	0.074 (− 0.034–0.182)
Multi person household	−0.062 (− 0.139–0.015)	−2.928 (− 8.900–3.044)	−0.002 (− 0.070–0.066)	−0.004 (− 0.104–0.096)
Ventilation	− 0.045 (− 0.177–0.088)	−4.228 (− 14.446–5.990)	−0.023 (− 0.140–0.094)	−0.032 (− 0.204–0.141)
Injury^2^				
Head or face	0.080 (−0.010–0.169)	10.884 (3.982–17.786)*	0.083 (0.003–0.162)*	0.077 (−0.039–0.193)
Thorax	0.069 (−0.006–0.144)	8.157 (2.371–13.943)*	0.094 (0.028–0.161)*	0.106 (0.009–0.203)*
Abdomen	0.138 (0.012–0.264)*	7.969 (−1.768–17.706)	0.099 (−0.013–0.212)	0.160 (−0.006–0.326)
Spine	−0.025 (− 0.130–0.080)	3.581 (−4.529–11.692)	−0.025 (− 0.119–0.068)	−0.033 (− 0.171–0.106)
Extremity	− 0.031 (− 0.122–0.060)	4.042 (− 2.953–11.036)	−0.013 (− 0.094–0.067)	−0.015 (− 0.133–0.104)
ISS	− 0.010 (− 0.017– − 0.004)*	−0.803 (− 1.327– − 0.280)*	−0.008 (− 0.014– − 0.002)*	−0.015 (− 0.024– − 0.006)*
Pre-injury EQ-VAS	–	0.362 (0.177–0.548)*	0.002 (− 0.000–0.004)	0.002 (− 0.001–0.005)
Pre-injury EQ-5D	0.487 (0.289–0.686)*	–	–	–
H-LOS	−0.005(− 0.008– − 0.002)*	−0.335 (− 0.567– − 0.103)*	−0.005 (− 0.008– − 0.003)*	−0.007 (− 0.011– − 0.003)*
GCS	−0.009 (− 0.021–0.003)	−0.576 (− 1.512–0.360)	−0.002 (− 0.012–0.009)	−0.002 (− 0.017–0.014)

Legend: Longitudinal analysis adjusted for time. *EQ-5D* Euroqol-5D, *HUI* Health Utilities Index, Beta Regression coefficient, *CI* Confidence interval, *ASA* American Society of Anaesthesiologists classification, *ISS* Injury Severity Score, *H-LOS* Hospital length of stay, *GCS* Glasgow Coma Scale. *p < 0,05; ^1^ASA I is the reference category; ^2^Patients that had at least an injury severity ≥ 3

#### Mobility

One week after trauma 83.5% (*N* = 71) of the patients had mobility problems and 12 months after trauma 44.1% (*N* = 75) of the patients had mobility problems. After adjustment for confounding H-LOS (OR = 1.69; 95% CI: 1.20–2.39) and an extremity injury (OR = 2.77; 95% CI: 1.36–5.62) were found to be significant prognostic factors for impairments in mobility.

#### Self-care

Problems in self-care were reported in 78.6% (*N* = 66) and 15.3% (*N* = 26) of the patients one week and 12 months after trauma respectively. An ASA III or ASA IV classification (OR = 3.16; 95% CI: 1.48–6.74) and hospital length of stay (OR = 1.50; 95% CI: 1.16–1.93) were significant prognostic factors for self-care problems in the adjusted model. Patients with an abdomen injury showed significant less self-care problems (OR = 0.34; 95% CI: 0.13–0.89).

#### Activity

Problems with daily activities were reported in the one week after trauma period by 89.4% (*N* = 76) of the patients and 12 months after trauma by 46.4% (*N* = 78) of the patients. H-LOS (OR = 1.55; 95% CI: 1.09–2.19) was the only significant adjusted prognostic factor for activity problems during one year after trauma.

#### Pain

One week after trauma 87.2% (*N* = 75) of the patients reported pain and 12 months after trauma 53.3% (*N* = 89) of the patients reported pain. In the adjusted model pre-injury pain complaints (OR = 1.93; 95% CI: 1.00–3.73), a spine injury (OR = 2.42; 95% CI: 1.08–5.42) and a multi person household (OR = 2.05; 95% CI: 1.21–3.46) were prognostic factors for pain impairments after trauma.

#### Anxiety

The percentage of patients reporting anxiety and depression problems remains stable*,* 31.4% (*N* = 27) one week after trauma and 32.5% (*N* = 55) twelve months after trauma. Pre-injury anxiety or depression (OR = 2.00; 95% CI: 1.35–6.63) was the only adjusted prognostic factor for anxiety or depression problems.

#### Cognition

**Fig. 3:**
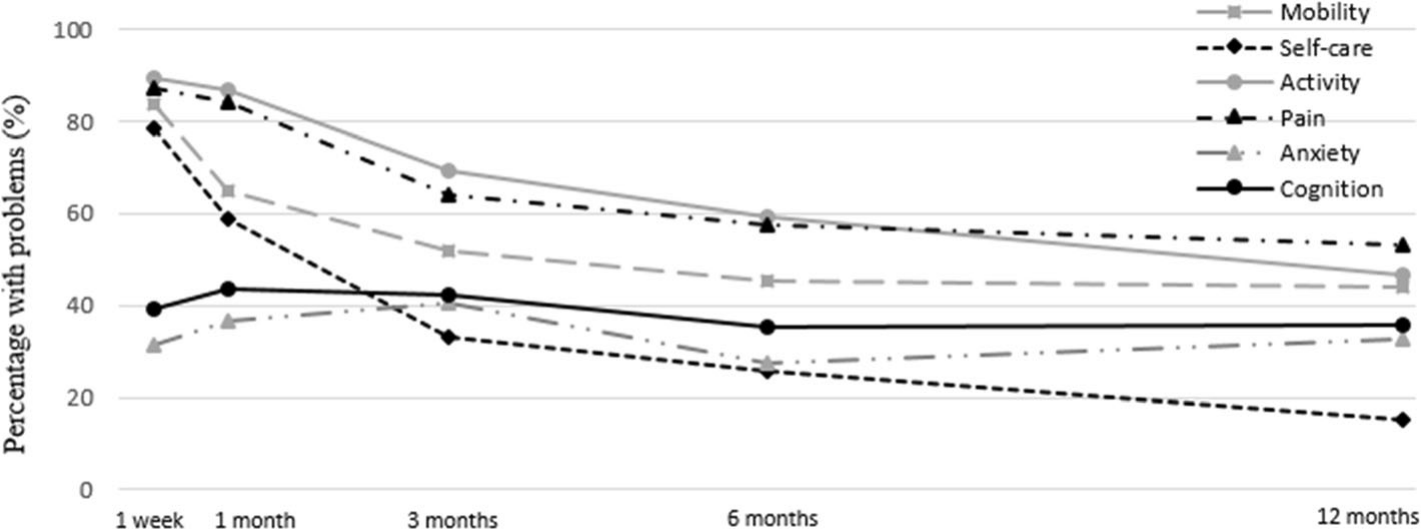
The percentage of patients with impairments in the EQ-5 dimensions and cognition impairments

One week after trauma 39.3% (*N* = 33) of the patients had problems with cognition and 12 months after trauma 35.7% (*N* = 60). Patients with a high educational level showed less cognition problems in the adjusted model (OR = 0.38; 95% CI: 0.17–0.86). See Fig. 3 and Table 3 for an overview of the percentage impairments and the odds ratios in the five dimensions of the EQ-5D and cognition problems.

**Table 3:** Problems in the five EQ-5 dimensions and cognition during one year after a severe injury

	Mobility OR (95% CI)	Self-care OR (95% CI)	Activity OR (95% CI)	Pain OR (95% CI)	Anxiety OR (95% CI)	Cognition OR (95% CI)
Ventilation	0.94 (0.52–1.72)	–	1.40 (0.73–2.68)	–	1.39 (0.68–2.82)	1.79 (0.86–3.74)
Female	1.19 (0.71–2.00)	–	1.46 (0.75–2.82)	–	1.88 (0.97–3.67)	–
Age	–	–	–	–	0.75 (0.52–1.08)	–
ISS	1.21 (0.84–1.74)	1.31 (0.92–1.85)	1.13 (0.74–1.72)	–	1.16 (0.84–1.60)	1.08 (0.71–1.63)
ASA II^1^	1.18 (0.64–2.17)	1.05 (0.62–1.77)	–	–	–	–
ASA III/IV^1^	2.22 (0.84–5.86)	3.16 (1.48–6.74)*	–	–	–	–
Middle educational level^2^	–	–	–	–	–	0.83 (0.43–1.59)
High educational level^2^	–	–	–	–	–	0.38 (0.17–0.86)*
H-LOS	1.69 (1.20–2.39)*	1.50 (1.16–1.93)*	1.55 (1.09–2.19)*	1.15 (0.88–1.50)	1.01 (0.74–1.37)	1.35 (0.96–1.86)
Pre-injury	2.25 (0.92–5.48)	1.93 (0.43–8.64)	2.45 (0.94–6.37)	1.93 (1.00–3.73)*	2.00 (1.35–6.63)*	2.39 (0.71–8.02)
Injury^3^						
Head/face	0.67 (0.34–1.33)	0.78 (0.40–1.55)	0.67 (0.34–1.32)	0.89 (0.51–1.57)	–	1.60 (0.71–3.61)
Thorax	0.66 (0.34–1.30)	0.61 (0.34–1.11)	0.71 (0.36–1.39)	0.87 (0.50–1.50)	–	0.63 (0.31–1.27)
Abdomen	0.53 (0.19–1.51)	0.34 (0.13–0.89)*	0.52 (0.17–1.58)	0.54 (0.23–1.24)	–	1.62 (0.48–5.54)
Spine	1.65 (0.74–3.72)	1.05 (0.48–2.32)	1.49 (0.59–3.76)	2.42 (1.08–5.42)*	–	0.51 (0.17–1.51)
Extremity	2.77 (1.36–5.62)	1.49 (0.73–3.03)	1.59 (0.73–3.47)	1.58 (0.79–3.12)	–	0.66 (0.28–1.54)
Employed	0.63 (0.38–1.06)	–	–	–	–	–
Multi person Household^4^	–	–	–	2.05 (1.21–3.46)	–	–

Legend: Longitudinal analysis adjusted for time. *OR* Odds Ratio, *CI* Confidence interval, *ISS* Injury Severity Score, *ASA* American Society of Anaesthesiologists classification, *H-LOS* Hospital length of stay, *GCS* Glasgow Coma Scale. *p < 0,05; ^1^ ASA I is the reference category; ^2^Low educational level is the reference category; ^3^Patients that had at least an injury severity ≥3; ^4^Single person household is the reference category

The unweighted percentage of problems in the Dutch reference population was 18.2% in mobility, 3.8% in self-care, 14.8% in activity, 34.4% in pain, 11.5% in anxiety and 8.4% in cognition.

### Health status: EQ-VAS

In the multivariable model a lower pre-injury EQ-VAS score was the only patient related prognostic factor over time for a lower EQ-VAS score (β = 0.362; 95% CI: 0.177–0.548). Trauma related prognostic factors over time were a higher ISS score (β = − 0.803; 95% CI: − 1.327– − 0.280) and a longer H-LOS (β = − 0.335; 95% CI: − 0.567– − 0.103). Patients with a head injury (β = 10.884; 95% CI: 3.982–17.786) and patients with a thorax injury (β = 8.157; 95% CI: 2.371–13.943) showed a higher EQ-VAS score compared to patients without these injuries. See Table 2 for an overview of the prognostic factors.

### Health status: HUI2

Comorbidities (an ASA III or ASA IV classification; β = − 0.176; 95% CI: − 0.289– − 0.063) was the only patient related prognostic factor for a lower HUI2 utility score over time, adjusted for confounding factors. Adjusted trauma related prognostic factors over time were longer H-LOS (β = − 0.005; 95% CI: − 0.008– − 0.003) and a higher ISS (β = − 0.008; 95% CI: − 0.014–0.002). Patients with a head injury or a thorax injury showed a significant higher HUI2 score compared to patients without these injuries. See Table 2 for an overview of the prognostic factors.

### Health status: HUI3

For the HUI3 score the only patient related prognostic factors over time in the multivariable model were ASA III or ASA IV classifications (β = − 0.213; 95% CI: − 0.376– − 0.049). Trauma related prognostic factors over time were a higher ISS score (β = − 0.015; 95% CI: − 0.024– − 0.006) and a longer H-LOS (β = − 0.007; 95% CI: − 0.011– − 0.003). Patients with a thorax injury showed a higher HUI3 score (β = 0.106; 95% CI: 0.009–0.203) compared to the patients without a thorax injury. See Table 2 for an overview of the prognostic factors.

### Prognostic factors over time

An overview of the effects of all variables on the different time points is shown in Additional file 1.

A higher ISS and longer H-LOS were both short- and long-term prognostic factors for a lower health status after trauma and the effect of both decreased over time.

A younger age and an extremity injury were short term prognostic factors for a lower health status after a severe trauma. Patients with a head injury showed a higher health status compared to patients without this type of injury during the first months after trauma.

Unemployment before trauma and comorbidities (ASA III or ASA IV) were long-term prognostic factors for a lower health status. The patients with an ASA II classification showed the same trend as patients with an ASA III or ASA IV classification, although not significantly lower compared to patients with an ASA I classification. Patients with an abdomen or a thorax injury showed a higher long-term health status.

## Discussion

In our study, patients reported a lower health status in the first year after injury compared to their pre-injury score. The largest increase of health status was found in the first months after trauma. Between three and six months after trauma health status increased slightly and between six and twelve months it remained almost stable. Prognostic factors for a lower health status during twelve months after a severe trauma were a lower pre-injury health status, longer H-LOS, higher ISS and a higher ASA classification. A younger age and an extremity injury were prognostic factors during the first months after a severe trauma. Unemployment before trauma was a prognostic factor for a lower health status from six months after trauma, probably caused by a higher adaptability in the employed population compared to the unemployed population. Health status depends on both patient and trauma characteristics including injured body area and the severity of the injury. These results are consistent with comparable international studies [20–24]. However, educational level [22] and a single person household [24] were no prognostic factors for a lower health status in this study.

The largest decline in percentage of patients with problems in the EQ-5 dimensions was found between one month and three months after trauma. However, even twelve months after a severe trauma our population reported more problems in all dimensions compared to the Dutch reference population [30]. Most prevalent problems were found in the EQ-5 dimensions mobility, self-care, activity and pain. It is consistent with comparable studies that these physical dimensions are more negatively affected than mental dimensions, especially during the first months after trauma [47, 48]. Problems reported in these dimensions decreased over time. The dimensions of anxiety and cognition remain almost equal twelve months after trauma. The pre-injury score of pain and anxiety were significant prognostic factors in pain and anxiety dimensions, trauma appears to be a precipitating factor to develop anxiety and depression post-injury. An extremity injury is an important prognostic factor for mobility problems, probably as a result of the inability to walk. For many extremity injuries weight-bearing is allowed four until six weeks after trauma, from then on the mobility of the patients will improve considerably until six months after trauma. This is in line with the lower health status until one month after trauma found in patients with an extremity injury.

An interesting and unexpected result is the higher health status until one month after trauma in patients with a head injury compared to patients without a head injury. A head injury is associated with significant early limitations in most aspects of everyday life [49]. However, this phenomenon is also shown in other studies [50, 51], in which the disease-specific health-related quality of life revealed no differences one year after a traumatic brain injury. This could be a result of recalibration, reconceptualization and reprioritization of internal standards and references utilized for self-appraisal, also called response shift [52].

A comparable phenomenon is seen in the patients without comorbidities (ASA I). These patients are, as far as we know, healthy, but in the first months after trauma they showed a lower health status compared to the patients with moderate comorbidities (ASA II). Probably based on slower recalibration or reprioritization in the relatively healthy patients during the first period after trauma, because they have never recalibrated or reprioritized before. The trend over time of the patients with an ASA III or ASA IV classification is just as expected based on their lower recovery possibilities because of their comorbidities. This is in line with the results of Tran et al. (2017) [53] who showed that pre-injury ASA score is an independent predictor of readmission after a major injury, when assumed that this also leads to a lower health status.

Patients with severe abdominal or thoracic injuries showed a higher health status over time compared to patients without these injuries. Most of these injuries could be life-threatening (e.g. massive blood loss) and require acute treatment. However, if the patients survives the effect on health status during the first months after trauma is limited and after three months they reported a higher health status compared to patients these injuries.

Based on previous research ICU admission or ICU length of stay was expected to be a prognostic factor for health status over time after a severe injury. However, in our population ICU admission or ICU length of stay was not a prognostic factor for health status. Probably ICU admission or ICU length of stay is more related to the functional outcome [21] instead of health status. The relationship between functional outcome and health status is unknown.

### Study limitations

A limitation of this study could be the inclusion rate of 55%. It is unknown whether these patients have a poor, good or equal health status compared to the patients who did not participate in this study. However, the non-responders had significant more patients with an ASA III or ASA IV classification. Probably, these patients are not able to respond because of their health problems. Therefore, health status and the effect of the comorbidities on health status could be underestimated in our population. Besides, within the patients with a head injury the lowest response rate was found one week after trauma. Maybe the patients with severe disabilities caused by their head injury did not responded to the questionnaires.

Secondly, maybe these generic questionnaires (EQ-5D and HUI) are not sensitive enough to recognize disabilities caused by a head or face injury. Specific questionnaires for severely injured patients or patients with a head or face injury are the Glasgow Outcome Scale Extended (GOSE) or Quality of Life after Brain Injury (QOLIBRI). It could be that the higher health status found during the first month will not be found when using the GOSE or QOLIBRI.

Thirdly, health status could be influenced by many different factors and maybe not all of these factors are measured in our study. However, many possible confounders are measured and they were tested both in a uni- and multi-variable model.

Last, proxy and patient questionnaires are analysed equally but health status may differ in proxy and patient responses. Gabbe et al. (2012) [54] showed that the differences show a random variability rather than systematic bias. Therefore, proxy questionnaires could suffer from bias when assessing individual patient recovery, but they are unlikely to bias group comparisons.

Based on the provided insight into health status and prognostic factors over time twelve months after a severe trauma, clinicians should be aware of patients at risk for a lower health status at different time points. Patients with multiple negatively prognostic factors should derive a multidisciplinary approach. Further research should attempt to analyse if a multidisciplinary approach improves health status in these patients.

## Conclusions

One year after a severe trauma patients reported more problems in all health status dimensions compared to the reference population in our country. A lower pre-injury health status, longer H-LOS, higher ISS and comorbidities are important prognostic factors for a lower health status one year after a severe injury. There are different short- and long-term prognostic factors.

## Supplementary information

**Additional file 1.** The regression coefficient of the multivariable linear mixed model are visualised, adjusted for all other prognostic factors.

## Data Availability

The datasets generated and analysed during this study are not publicly available due to privacy reasons but are available from the corresponding author (R.J.M. Havermans) on reasonable request.

## Abbreviations

AIC: Akaike Information Criterion
AIS: Abbreviated Injury Scale
ASA: American Society of Anaesthesiologists
BIOS: Brabant Injury Outcome Surveillance
EQ-5D: EuroQol-5D
EQ-VAS: EQ Visual analogue scale
GOSE: Glasgow Outcome Scale Extended
H-LOS: Hospital length of stay
HRQoL: Health related quality of life
HS: Health Status
HUI: Health Utilities Index
ICU: Intensive Care Unit
ISS: Injury Severity Score
MICE: Multivariate imputation by chained equations
QOLIBR: Quality of Life after Brain Injury
