# Supplementary material for: Longitudinal analysis of health status the first year after trauma in severely injured patients
Source: Scand J Trauma Resusc Emerg Med. 2020 Apr 20;28:29. doi: 10.1186/s13049-020-00719-8 (PMC7169038; doi:10.1186/s13049-020-00719-8)

**Additional file 1 -** The regression coefficient of the multivariable linear mixed model are visualised, adjusted for all other prognostic factors. The regression coefficient on each time point is calculated by changing the reference category of the time variable.

Pre-injury HS: The EQ-5D-3L utility score is used for the EQ-5D-3L utility score and the pre-injury EQ-VAS score is used for the HUI2 and HUI3 utility scores and the EQ-VAS score.


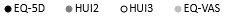

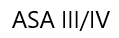

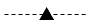

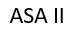

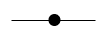

Supplement: Supplementary file 1 — Additional file 1. The regression coefficient of the multivariable linear mixed model are visualised, adjusted for all other prognostic factors. [file 13049_2020_719_MOESM1_ESM.docx]
